# Functionalities of Octenyl Succinic Anhydride Wheat Starch and Its Effect on the Quality of Model Dough and Noodles

**DOI:** 10.3390/foods14101688

**Published:** 2025-05-10

**Authors:** Hongxue Ma, Liai Yang, Dunhe Zhang, Huijing Chen, Jianquan Kan

**Affiliations:** 1 College of Food Science, Southwest University, 2 Tiansheng Road, Beibei, Chongqing 400715, Chinaswu2639023@email.swu.edu.cn (L.Y.); 17390501275@163.com (D.Z.); 2 Chinese-Hungarian Cooperative Research Centre for Food Science, Chongqing 400715, China; 3 Laboratory of Quality & Safety Risk Assessment for Agri-Products on Storage and Preservation (Chongqing), Ministry of Agriculture and Rural Affairs of the People’s Republic of China, Chongqing 400715, China

**Keywords:** wheat starch, octenyl succinic anhydride, modification, model dough, application

## Abstract

Chemically modified starch is a widely used food additive for tailoring the quality of wheat flour products. However, the effects of octenyl succinic anhydride (OSA)-modified wheat starch with varying degrees of substitution on the quality of dough and noodles remain unclear. In this study, we prepared two types of OSA-modified wheat starch with different degrees of substitution and incorporated them as additives into a wheat starch–gluten protein model flour system to evaluate their impact on dough processing characteristics. Fourier transform infrared (FTIR) spectroscopy results revealed the introduction of ester carbonyl (C=O) and carboxylate (RCOO−) functional groups into the starch structure. X-ray diffraction (XRD) analysis demonstrated that OSA modification reduced the relative crystallinity of starch and disrupted the long-range structural order of the native starch. Scanning electron microscopy (SEM) observations indicated that the surface of OSA-modified wheat starch granules became rougher. OSA modification enhanced the solubility, water absorption capacity, and apparent viscosity but lowered the gelatinization temperature of starch, making starch more prone to gelatinization. Furthermore, the incorporation of OSA-modified wheat starch significantly altered the gelatinization behavior and dynamic rheological properties of wheat dough, whilst the noodle with the addition of OSA-modified starch (DS = 0.019) reduced the cooking time by 29.0% compared to the control group noodle and improved its water absorption rate. This study provides a theoretical foundation for the application of OSA-modified wheat starch as a food additive in wheat-based foods.

## 1. Introduction

Wheat starch is the most abundant component in wheat flour, which serves as a raw material for processing into various widely consumed food products such as noodle, bread, and biscuit [1]. Therefore, the rational regulation of starch-processing characteristics is crucial for manufacturers aiming to produce high-quality flour-based foods. Currently, a wide range of improvers, including non-starch polysaccharides, emulsifiers, and starch derivatives are widely used to enhance the physical and chemical properties of wheat-based foods. These additives help meet the diverse quality expectations of consumers for wheat-based foods [2,3]. For example, Li et al. [4] investigated the effects of hydrocolloids from various sources on the rheological and fermentative properties of dough. Their findings revealed that linear-chain hydrocolloids with higher viscosity significantly enhanced the gluten network. Considering that OSA-modified starch has been shown to improve the water retention and texture characteristics of baked products, Balic et al. [5] investigated the effectiveness of OSA-modified wheat and cassava starch as fat substitutes in bread formulations and found that bread samples containing 4% OSA-modified wheat and cassava starch can be used as fat substitutes in bread production. Generally, noodles have a status equivalent to rice as a staple food and are a highly popular starch-based food in Asia and many other regions. They account for 20–50% of the total wheat consumption in Asia [6]. However, with the accelerating pace of modern life, convenience foods that can be prepared and consumed quickly have become the preferred dietary choice for many office workers. Consequently, the current challenge lies in finding straightforward methods for producing noodles that can be cooked quickly while maintaining the desired eating qualities. This could further enhance the consumption of wheat flour and noodles.

Starch serves as an essential component of wheat-based foods and provides desirable physicochemical and sensory attributes, such as texture, mouthfeel, and appearance [7]. Consequently, regulating the functional properties of wheat starch is critical to achieve diverse qualities in wheat-based products. Native starch (starch without secondary treatment) exhibits limited functionality, owing to its relatively rigid internal structure, including low solubility, poor freeze–thaw stability, weak shear and heat resistance, susceptibility to enzymatic hydrolysis, rapid rehydration, and high digestibility. These limitations hinder the ability to meet market application demands fully. However, the presence of reactive hydroxyl groups in the structural units of starch provides opportunities for chemical reactions with modifiers, thereby enabling structural adjustments that alter its functionality [8]. As a result, various types of modified starches produced through different technologies are widely utilized in both food and non-food industries (e.g., papermaking and pharmaceuticals). Starch is typically modified using derivatization methods such as etherification, esterification, cross-linking, and starch grafting. These derivatives have garnered significant attention in food and other industries [9,10]. Among these, anhydride-modified starch derivatives are a common category of chemically modified starch with enhanced properties. The starch structure is typically modified using dicarboxylic anhydrides or organic acid esters. The hydrophobic groups of the modifier undergo nucleophilic substitution with hydroxyl groups on the native starch structural units, introducing both hydrophilic and hydrophobic groups into the starch molecules. This modification endows starch with excellent emulsifying properties while altering its gelatinization and retrogradation behaviors [11]. Octenyl succinic anhydride (OSA), a dicarboxylic anhydride, is the only acidic anhydride approved for use as a food additive. It has been extensively applied in the functional modification of starch and non-starch polysaccharides. Thus, OSA-modified starch is a safe food ingredient that can be incorporated into wheat-based foods. However, the specific effects of adding OSA-modified wheat starch to wheat flour on the processing characteristics of wheat dough and the quality of the resulting baked products remain unclear.

Considering the aforementioned circumstances, to enhance the physicochemical properties of native starch and its utilization value, as well as the cooking properties of wheat noodles in this study, OSA was employed as a modifier to prepare two types of OSA-modified wheat starch with different degrees of substitution. The effects of varying esterification levels on the structure and properties of wheat starch were analyzed using FTIR, XRD, and rapid viscosity analyzer (RVA). The OSA-modified starches were then incorporated into wheat flour as food improvers to further investigate their impact on the rheological properties of wheat dough and the cooking characteristics of noodles. The results of this study may provide deeper insights into the application of OSA-modified wheat starch in flour-based products, thereby enhancing the application value of wheat starch in the food industry.

## 2. Materials and Methods

### 2.1. Materials

Wheat starch was isolated from wheat flour (87.83% starch, 0.21% protein, 0.18% fat) purchased from Ningxia SaiBeiXue Flour Co., Ltd. (Ningxia, China); OSA was obtained from Shenzhen Silikai Trading Co., Ltd. (Shenzhen, China); and ethanol was sourced from Chongqing Chuandong Chemical (Group) Co., Ltd. (Chongqing, China). The water used in the experiment was distilled, and all other chemical reagents were of analytical grade.

### 2.2. Wheat Starch Extraction and Preparation of Modified Starch

(1) Wheat starch extraction

A mixture of wheat flour and water was prepared in a 2:1 ratio (*w*/*v*) to form a dough. The formed dough was washed with water to completely remove the starch. The starch–water mixture was centrifuged, and the solid layer was separated, dried, and filtered through a 100-mesh sieve.

(2) Preparation of modified starches

OSA-modified wheat starch samples were prepared using the method of Xu et al. [12]. Briefly, wheat starch (100 g) was mixed with deionized (DI) water to prepare a starch suspension with a mass fraction of 30%. The pH of the starch suspension was adjusted to 8.0 using a NaOH solution (3% *w*/*v*); then, the OSA (3% and 5% *w*/*w* of the dry weight of the wheat starch) was diluted with anhydrous ethanol (OSA/anhydrous ethanol = 1/4, *v*/*v*) and added dropwise to the starch suspension. The reaction temperature and reaction time were 45 °C and 3 h, respectively. During the reaction, the pH of the reaction system was adjusted to 7.5–8.0 using a NaOH solution. After the reaction, the reactants were centrifuged (3500× *g*) for 10 min. The precipitate was washed three times with a 75% ethanol solution and then once with DI water. After centrifugation, drying, grinding, and filtering through a 100-mesh sieve, two wheat starches modified with OSA were obtained, labeled as EWS1 and EWS2, respectively. The unmodified starch was named CWS.

### 2.3. Determination of Degree of Substitution (DS)

The DS was determined according to the method of Chen et al. [13], with minor modifications. Briefly, starch (1.0 g) was mixed with 5 mL of isopropanol and magnetically stirred at 500 r/min for 10 min. Subsequently, 15 mL of 2.5 M HCl–isopropanol solution was added, and the solution was stirred for an additional 30 min. After filtering, the wheat starch sample was washed repeatedly with DI water until no chloride remained (silver nitrate solution was used as an indicator). Finally, 200 mL of DI water was added to the washed wheat starch sample and heated in a boiling water bath for 20 min. Immediately, the wheat starch sample solution was titrated with 0.05 M NaOH solution until the pink endpoint, indicated using phenolphthalein. DS can be calculated using Equation (1):

(1) $$DS=\frac{0.162\times \frac{A\times C}{W}}{1-\left[0.210\times \frac{A\times C}{W}\right]}$$

where 162 and 210 are the molar masses of the glucose residues and octenyl succinic anhydride, respectively; *A* represents the volume of NaOH standard solution required to titrate the wheat starch samples (mL); *C* is the molar concentration of the NaOH solution (0.02 M); and *W* is the mass of the wheat starch samples (g).

### 2.4. Fourier Transform Infrared (FT-IR) Spectroscopy

Adopting the method of Chen et al. [14], starch samples (5 mg) were mixed with dried potassium bromide (500 mg) and ground and pressed into thin pellets. The functional group changes in the starch molecular structure were analyzed using Fourier transform infrared spectroscopy (Spectrum100, PerkinElmer, Shelton, CT, USA). The wavenumber range of 4000–500 cm^−1^ was scanned 32 times at a resolution of 4 cm^−1^.

### 2.5. X-Ray Diffraction (XRD)

Starch was evenly spread in the sample cell, and the crystalline structure of the sample was determined using X-ray diffractometer (XRD; X’Pert3 Powder, Malvern Panalytical, Almelo, The Netherlands). The diffraction angle scan range was 5–45°, the scanning speed was 2°/min, the step size was 0.05°, and the accelerating voltage and current were 40 kV and 40 mA, respectively. The relative crystallinity (*RC*) was determined using Equation (2):

(2) $$RC\left(\%\right)=\frac{A_{c}}{A_{a}+A_{c}}\times 100\%$$

where *A_c_* and *A_a_* represent the crystalline and amorphous areas, respectively.

### 2.6. ^1^H Nuclear Magnetic Resonance (NMR) Spectroscopy

The starches (20 mg) were completely dissolved in DMSO-d_6_ (1.0 mL) while heating in a 90 °C thermostatic water bath. The ^1^H NMR spectra of the starch sample were obtained over 256 scans using a nuclear magnetic resonance spectrometer (Bruker Avance III 400 M, Karlsruhe, Germany).

### 2.7. Scanning Electron Microscopy (SEM)

The wheat starch samples were evenly dispersed on the surface of the conductive adhesive and sprayed with gold. Their particle surface morphologies were observed using a scanning electron microscope (Phenom Pro10102, Eindhoven, The Netherlands) with an accelerating voltage of 10 kV.

### 2.8. Determination of Solubility and Swelling Degree

Solubility and swelling degree were determined using the method of Xu, Zhao, and Chen [15], with minor modifications. First, 0.5 g of the starch samples were mixed with 30 mL of DI water. The mixture was placed in a 90 °C water bath for 30 min; then, the starch samples were centrifuged (3500× *g*) for 10 min. The precipitate was weighed, and the supernatant was dried at 105 °C for 24 h and weighed. The solubility and swelling degree of the wheat starch samples were calculated using Equations (3) and (4):

(3) $$Solubility\;in\;water\;\left(\%\right)=\frac{weight\;of\;dry\;starch\;supernatant}{weight\;of\;starch\;sample}$$

(4) $$\mathrm{Swelling}\;\mathrm{power}\;(g/g)=\frac{\mathrm{weight}\;\mathrm{of}\;\mathrm{starch}\;\mathrm{sediment}}{\mathrm{weight}\;\mathrm{of}\;\mathrm{starch}\;\mathrm{sample}\times (1-\mathrm{Solubility}\;\mathrm{in}\;\mathrm{water})}$$

### 2.9. Determination of Pasting Properties

According to the method of Wen et al. [16], 2.0 g of starch samples was weighed into aluminum sample canisters. Then, 26 mL of DI water was added to each sample and stirred evenly. The gelatinization characteristics of the wheat starch samples were measured using a Rapid Visco Analyzer (RVA; Model RVA-4, Warriewood, Australia); the samples were heated from 50 to 95 °C and finally cooled to 50 °C.

### 2.10. Determination of Dynamic Rheological Properties

After measuring the pasting characteristics of wheat starch paste (3.0 g), the sample was placed in the center of the stage of an MCR 302 rheometer (Anton Paar GmbH, Graz, Austria). Excess sample was carefully removed. A PP50 probe (d = 50 mm) was selected, and the preset distance was 2.0 mm. Silicone oil was applied around the edges of the wheat starch paste to prevent water evaporation during measurement. Dynamic oscillation frequency and temperature scanning were performed. The dynamic oscillation frequency was scanned at a constant temperature of 25 °C within the frequency range of 0.1–100 rad/s at a strain of 1%. For the dynamic oscillation temperature scan, the sample was scanned across a temperature range of 25–90 °C, maintaining a strain amplitude of 1% and a frequency of 1 Hz.

### 2.11. Mixolab Behavior of Model Flour

Dough samples were prepared according to a previously reported method [17]. Briefly, 12 g of CWS, EWS1, and EWS2 were each mixed with 74 g of wheat starch and 14 g of gluten to obtain the model dough flour. The flours and DI water were mixed in a Mixolab analyzer (Chopin, Villeneuve-la-Garenne Cedex, France), and the freshly prepared doughs were named CWSD, EWS1D, and EWS2D, respectively. After stirring for 8 min, the following procedure was carried out: the test temperature was initially increased from 30 to 90 °C at a rate of 4 °C/min, and the dough was stirred for 7 min; then, the temperature was decreased from 90 to 50 °C at a rate of 4 °C/min, with 5 min of stirring. Meanwhile, the C1, C2, C3, C4, and C5 values of the whole test process were recorded.

### 2.12. Cooking Characteristics Measurement

Cooking characteristics were measured according to a previously described method for noodles [18]. The uncooked dough prepared in Section 2.11 was used for preparing the noodles (20 cm in length); then, the noodles (20 g; m0) were boiled in 400 mL of water. Their cooking time was measured using a stopwatch to ensure that no raw sections remained. The cooked noodles were rinsed, drained, and weighed (m1). The ratio of m1 to m0 was considered the water absorption rate of the noodles. Cooking loss was defined as the ratio of the dry matter weight in the boiling water (m2) to the weight of the dried noodles (m0).

### 2.13. Statistical Analysis

All data are presented as the mean ± standard deviation (SD) and were analyzed using SPSS 26.0 software (IBM, Chicago, IL, USA). An analysis of variance (ANOVA) was carried out using Duncan’s multiple range test (*p* < 0.05).

## 3. Results and Discussion

### 3.1. Degree of Substitution

**Figure 1:**
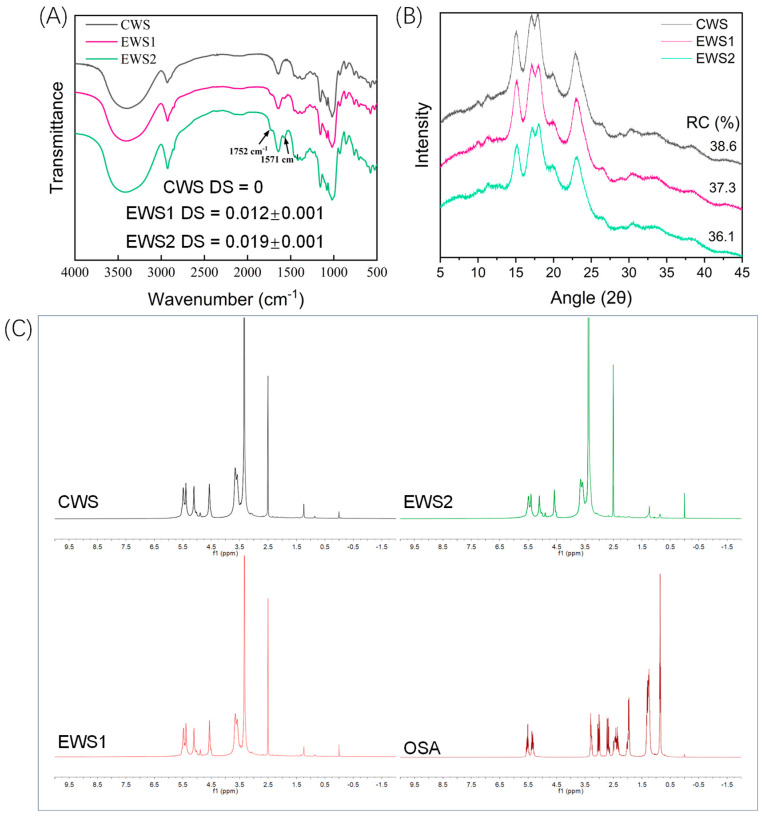
DS and FT-IR spectrum of wheat starch (**A**); XRD pattern (**B**); ^1^H NMR (**C**).

In theory, the hydroxyl groups at the C2, C3, and C6 positions of the D-glucopyranosyl unit of starch can be substituted by carboxyl groups or other functional groups. This substitution reaction primarily occurs within starch granules. The DS refers to the average number of hydroxyl groups replaced per glucose unit, which is directly related to the esterification degree of the substance [19]. The results in Figure 1A indicate that the DS of esterified starch increases with the increase in anhydride amount. The DS values of EWS1 and EWS2 were 0.012 and 0.019, respectively. Generally, OSA is insoluble in water; it tends to float on the surface of water in the form of large oil droplets within the aqueous-phase system. Consequently, it struggles to penetrate into the starch granules suspended in water. This limits the reaction site to the surface of the granules, leading to an uneven distribution of OSA groups within the starch particles. This uneven distribution also contributes to a relatively low DS between OSA and starch [20]. Generally, OSA starch has been approved as a food additive in a maximum amount of 3% and DS < 0.02. Producers of different enterprises can choose the OSA starch according to food safety standards and their own product requirements to regulate the quality of the final product.

### 3.2. FT-IR Spectroscopy Analysis

FT-IR spectroscopy is widely used to characterize the functional groups of starch. The IR spectra of different starch samples are shown in Figure 1A. The spectra reveal that the starch samples are similar, all displaying characteristic peaks associated with polysaccharides. For instance, the peak in the range of 3600–3000 cm^−1^ can be attributed to the O–H stretching vibration in starch, while the band at 1635 cm^−1^ may correspond to water absorption in the amorphous region of starch. Additionally, the absorbance peaks at 1045 cm^−1^ and 1022 cm^−1^ can be ascribed to the crystalline and amorphous structures of starch, respectively [21,22]. Notably, unlike native wheat starch, both types of esterified starches gave rise to two new characteristic absorption peaks at 1752 cm^−1^ and 1571 cm^−1^, which correspond to the stretching vibration of C=O and the asymmetric stretching vibration of RCOO^−^, respectively. The intensities of these two new peaks increased with rising DS value. This directly confirms that the esterification modification reaction between wheat starch and OSA successfully introduced new functional groups into the structure of starch molecules. Consequently, this triggers changes in the physicochemical properties of starch.

### 3.3. XRD Analysis

XRD analysis can be used to determine the long-range structural order of polymers, such as the crystalline structure of starch [23]. The XRD patterns in Figure 1B reveal the crystalline structures of the different wheat starch samples. All starches exhibited significant diffraction peaks at 2θ = 15.0°, 17.0°, 18.0°, 19.8°, and 23.0°. Additionally, we identified a weak absorption peak at ~20° for all wheat starches, indicating the presence of V-type structured starch–lipid complexes in the starch. This is due to the interaction between endogenous lipids and starch during storage. Therefore, the XRD patterns show that wheat starch possesses an A + V crystal structure [24]. At the same time, the OSA modification did not affect the intensity of this diffraction peak and did not alter the crystal structure of starch; however, modification reduced the RC of starch, and when the DS was 0.019, the RC decreased from 38.6% to 36.1%, which was similar to our previous report [25].

### 3.4. ^1^H NMR Spectroscopy

The solution ^1^H NMR spectra of different starch samples were acquired (Figure 1C). Owing to the similarity of the structural units, all starches show proton signals typical of starch. Specifically, the peaks at 0 ppm and 2.5 ppm in all the spectra correspond to the tetramethylsilane signal in the chemical reagents and the residual dehydrogenated DMSO signal, respectively. The chemical shifts at 5.3, 5.5, and 4.5 ppm are attributed to the OH-2, OH-3, and OH-6 protons of starch, while the signals at 5.1 and 3.1–3.6 ppm are assigned to H-1 and H-2, H-3, H-4, and H-5 in starch. These assignments agree with those reported by Meng et al. [26]. Compared with CWS, OSA-modified starch did not produce a distinct peak, owing to its low DS; as a result, the OSA groups were not readily detectable. The signal at 0.80–0.89 ppm is related to three protons in the terminal methyl group of OSA [27]. Notably, this signal for OSA-modified starch, particularly EWS2, is significantly stronger than that of the other two starches.

### 3.5. EM Analysis

**Figure 2:**
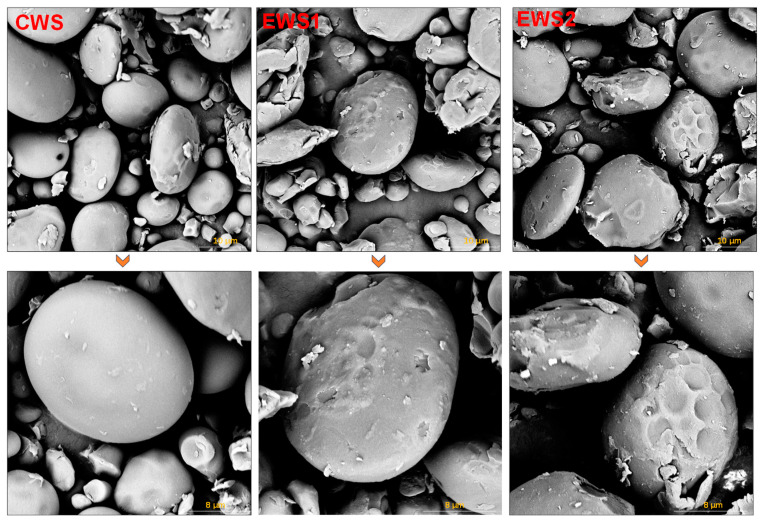
SEM images of wheat starch (magnification 5000× and 10,000×).

SEM provides valuable insights into polymer morphologies [28]. Figure 2 shows the morphology of starch as well as the influence of anhydride modification on the morphology of starch particles. Native wheat starch consists of flat, round, large particles and spherical small particles, with a smooth surface and high particle integrity. By contrast, after OSA chemical modification, the surface morphology of OSA-modified wheat starch particles changed significantly. This finding is consistent with the XRD patterns. Specifically, the surface of the starch particles transitioned from smooth and regular to rough, with noticeable depression and damage. Moreover, the higher the DS, the more pronounced the damage to the surface of OSA-modified wheat starch particles, which is consistent with the findings of Zhang et al. [29]. The damage to the starch particle morphology in OSA-modified wheat starch is attributed to erosion caused by anhydride and alkaline solutions on the surface of the starch particles during the OSA modification process. At the same time, these results indicate that the OSA esterification modification reaction may primarily occur on the surface of starch particles. Additionally, during wheat starch modification, the increase in broken and amorphous regions within the starch particles provides an accessible pathway for OSA to interact with the hydroxyl groups of wheat starch, thereby enhancing its esterification modification efficiency [30].

### 3.6. Solubility and Swelling Characteristics

**Figure 3:**
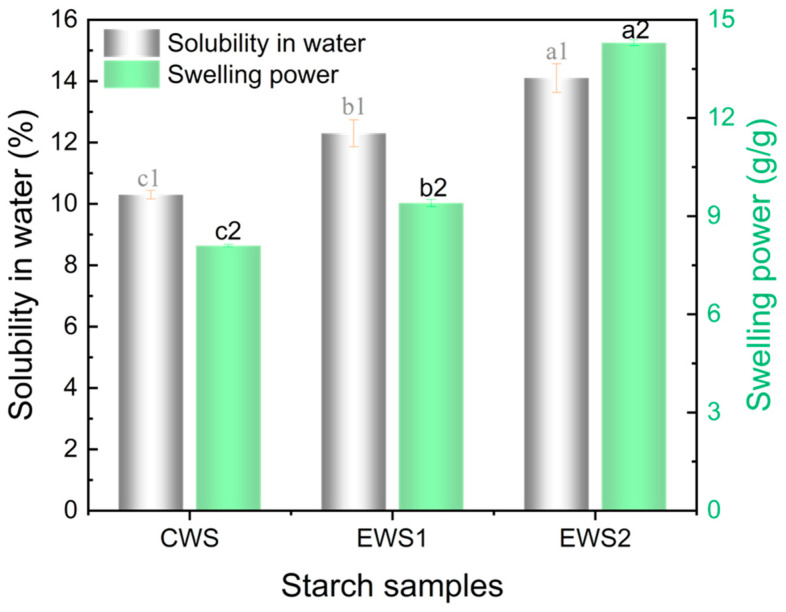
Solubility, and swelling power of starch samples. The same group of bars with different letters are significantly different (*p* < 0.05).

Most starches are semi-crystalline polysaccharides derived from plants. Owing to the abundant hydroxyl groups in their structures, starches are insoluble in cold water, and the swelling degree of ordinary starch in cold water is limited. Enhancing the solubility and swelling properties of starch can significantly improve its application potential [31]. The solubility and swelling characteristics of different starches are shown in Figure 3. The solubilities of CWS, EWS1, and EWS2 were 10.3%, 12.3%, and 14.1%, respectively, indicating that the solubility of OSA-modified wheat starch was higher than that of native wheat starch. Moreover, the solubility of wheat starch increased with increasing DS. This result may be attributed to the esterification reaction between wheat starch and OSA, which destroys the intact granular structure of starch (as shown in Figure 2), allowing water to more easily enter the interior. As a result, starch combines with more water molecules and then gelatinizes and disintegrates, thus increasing starch solubility. Furthermore, the results showed that the swelling degree of EWS2 was much higher than that of CWS. This indicates that the gelatinization behavior of the OSA-esterified modified starch changed and became more temperature-sensitive. This phenomenon can be explained by the introduction of bulky OSA groups into the starch structure through esterification, which disrupted the amorphous regions of the wheat starch and weakened the interactions between starch molecules. As a result, water molecules more easily penetrated the interior of wheat starch, thereby increasing the degree of swelling of the OSA-modified wheat starch.

### 3.7. Pasting Properties

**Figure 4:**
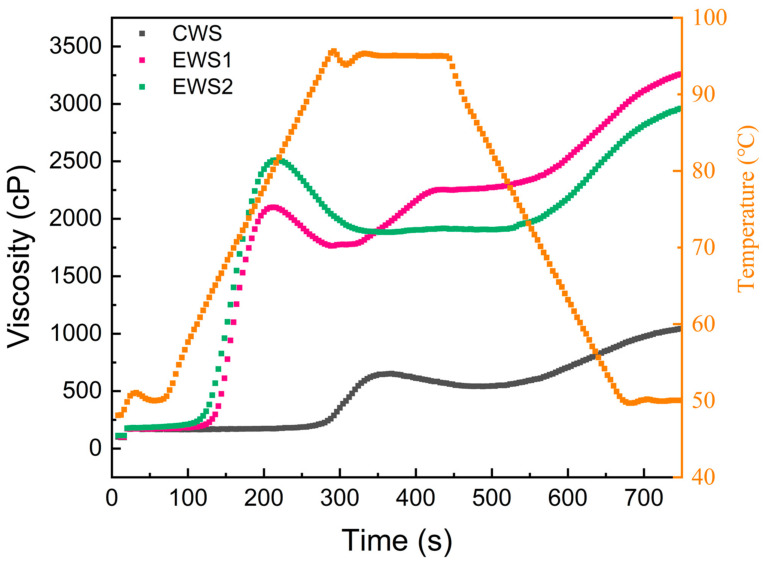
RVA profile of OSA wheat starch and mixed model flour.

RVA is widely used for studying the pasting properties of starch and involves monitoring the viscosity changes in a starch system based on rheological principles [32]. During heat treatment, the semicrystalline structure of the starch granules was disrupted. As the temperature increased, the starch gradually absorbed water and swelled, leading to double-helix dissociation, granule melting, particle morphology destruction, and amylose leaching. This resulted in the breakdown of the remaining short-range molecular order in the gelatinized starch, forming a viscous starch paste; this process is referred to as gelatinization [33]. The pasting profiles of different starch samples are shown in Figure 4. Throughout the gelatinization process, the viscosities of all starch samples tended to initially increase, then decrease, and finally increase again. As can be seen in Table 1, compared with the peak viscosity, breakdown viscosity, final viscosity, and setback viscosity of CWS (653, 112, 1044, and 503 cP, respectively), those of OSA-modified wheat starch were significantly higher, with a peak viscosity of 2239–2511 cP, breakdown value of 275–627 cP, final viscosity of 2960–3259 cP, and retrogradation viscosity of 1076–1295 cP. The increase in peak viscosity can be attributed to the conversion of some hydroxyl groups in wheat starch molecules into carboxyl groups through esterification. The introduction of OSA groups disrupted the amorphous structure of the starch molecules. During heating and gelatinization, modified starch readily absorbed water and expanded, promoting cross-linking between the starch molecular chains [34]. Additionally, higher breakdown values and setback viscosities indicate poorer thermal stability and shear resistance of the starch samples [35]. We also observed that after OSA modification, the gelatinization temperature of wheat starch was significantly lower than that of native wheat starch, indicating that starch becomes more sensitive to temperature and gelatinization. This phenomenon is likely due to the destruction of the starch morphology, which makes the internal structure of starch more susceptible to water molecule entry, as well as the introduction of chemical groups that disrupt the multiscale structure of starch and weaken the hydrogen bonding between starch molecules [36].

### 3.8. Mixolab Parameters of Mixed Flour

Mixolab analyzer was used to detect, the characteristics of gluten proteins under variable temperature mixing, the gelatinization and rehydration characteristics of starch during heating, and the synergistic effects among various cereal components under the dual action of stirring and heating. Meanwhile, Mixolab is widely used to evaluate the influence of flour and exogenous additives on its processing characteristics. Table 2 shows the effect of OSA starch on the Mixolab behavior of the dough. C1 represents the maximum torque during the initial mixing process and was highest for the model dough containing EWS2D. In the initial stage, the modified starch was more prone to gelatinization upon mixing with water, indicating that the addition of starch with a high DS can increase dough viscosity. After excessive mixing and heating, the torques of all the dough samples decreased, owing to the destruction of the gluten structure. C2 refers to the degree of dough strength loss when subjected to thermal–mechanical treatment, where a lower C2 value indicates stronger mechanical stability of the dough. The results show that the addition of modified starch reduced the C2 value of dough. This may be because OSA modification weakens the hydrogen bonds between starch molecules, making starch more sensitive to temperature and more prone to gelatinization. The increased viscosity leads to an increase in the dough’s torque during mixing. As the system temperature increased, gluten proteins and starch underwent complex reactions, such as aggregation or gelatinization, leading to an increase in the C3 value. C3, C4, and C5 represent the peak gelatinization viscosity of starch, viscosity maintained during gelatinization, and endpoint viscosity of starch recovery, respectively. Under moist conditions, starch began to gelatinize with increasing temperature, and the dough’s torque was mainly influenced by the degree of starch gelatinization and structural changes [37]. Additionally, C5 and C4 represent the rehydration characteristics of gelatinized starch during the cooling process. The addition of OSA-modified starch inhibited the rehydration of the model dough, indicating that a moderate amount of OSA starch can be used as a food additive to improve bread quality. This result highlights that moderate amounts of OSA starch can enhance the overall quality of bread products, potentially improving the texture and consistency.

### 3.9. Rheological Properties

#### 3.9.1. Dynamic Oscillation Frequency Sweep

**Figure 5:**
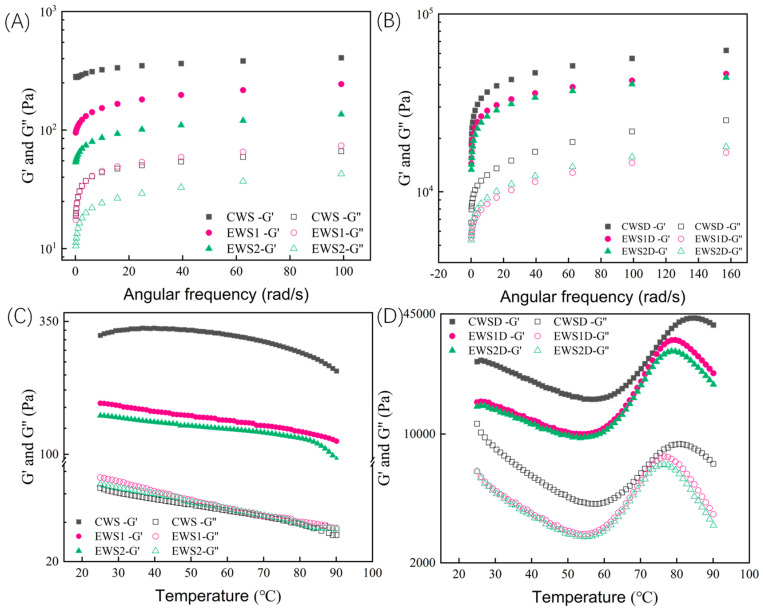
Frequency scans of starch samples (**A**) and model dough (**B**); temperature scans of starch samples (**C**) and model dough (**D**).

The energy storage modulus (G′), also known as the elastic modulus, reflects the energy stored in the material after oscillation, while the loss modulus (G″), also referred to as the viscous modulus, represents the energy dissipated during oscillation [38]. As can be seen in Figure 5A,B, both G′ and G″ of all the samples increased with the rise in oscillation frequency, and G’ was consistently higher than G″, indicating that the elastic properties dominated all the samples, showing typical solid-like characteristics. Compared with those of native wheat starch samples, both G′ and G″ of OSA-modified wheat starch generally exhibited a decreasing trend. This decrease may be attributed to the esterification modification reaction, which altered the molecular structure of the original wheat starch, causing the amylose in the amorphous region to dissolve and weakening the intermolecular forces between the starch molecules, thereby reducing G′ and G″ in the OSA-modified wheat starch samples [39]. As evident in Figure 5A, the G′ and G″ values of EWS2 were lower than those of CWS and EWS1, indicating that at this DS, the elasticity of the starch gel system was relatively weaker. The three-dimensional network gel structure loosened. Additionally, the incorporation of modified starch reduced the viscoelasticity of the model dough at varying frequencies (Figure 5B). Compared with CWSD, the addition of OSA-modified starch reduced its modulus. In the dough system, adding two types of starch with relatively low viscoelasticities could reduce the G′ and G″ values of the system to some extent, thus lowering the viscoelasticity of the dough. This effect may be related to changes in the structure of the gluten protein network within the system.

#### 3.9.2. Dynamic Oscillation Temperature Sweep

Figure 5C,D illustrate the viscoelastic properties of the starch samples and model dough in temperature-scanning mode. As heat-sensitive raw materials, starch and protein induce a series of complex chemical changes under heat treatment, and the resulting change in structure leads to a change in physical and chemical properties. The results revealed that the G′ values of modified starch samples were significantly lower than that of native wheat starch. This indicates that OSA modification weakened the solid-like characteristics of the starch gel system, which is consistent with the findings reported by Chen et al. [25]. As the measurement temperature increased, both the G′ and G″ values of native wheat starch and OSA-modified wheat starch samples exhibited an overall downward trend, with a more pronounced decrease in G′ for native wheat starch. This indicates that the gel network structure of wheat starch began to disintegrate at elevated temperatures and that OSA modification helped reduce the sensitivity of the gel network structure to temperature changes. Additionally, as shown in Figure 5D, with increasing measurement temperature, both G′ and G″ of the dough sample system initially decreased, increased, and finally decreased, which is the typical rheological behavior of dough during heating. This behavior may be attributed to the following sequence of events: initially, the rise in temperature caused the dough sample to soften, leading to a gradual decline in G′ and G″. As the temperature continued to increase, the starch granules swelled, expanded in volume, and formed a gel network structure, causing the G′ and G″ values of the dough to rapidly increase and reach their peak values. When the temperature exceeded 80 °C, the starch granules began to disintegrate, and the crystalline structure melted, resulting in a subsequent decrease in G′ and G″ of the dough sample. Notably, the G′ and G″ values of dough containing OSA-modified wheat starch were significantly lower than those of dough containing native wheat starch. This shows that the incorporation of OSA-modified wheat starch, which exhibited a lower viscoelastic modulus than native starch, reduced the viscoelasticity of the dough sample, indicating that the addition of OSA-modified wheat starch modulated the gelatinization behavior of the dough. We speculate that this phenomenon may be due to a weakening of the hydrogen bonds in starch upon OSA modification, thus reducing the viscoelasticity of the dough. Meanwhile, because hydrophobic long alkyl chains were introduced into the starch structure, a spatial hindrance effect occurred in the changed internal structure of starch, which may also reduce the interaction strength between starch and gluten.

### 3.10. Cooking Characteristics of Noodles

Starch is the primary component of noodles and plays a crucial role in noodle production and the regulation of the final noodle quality. With the quickening pace of modern lifestyles, noodles requiring shorter cooking times and exhibiting lower cooking loss rates have become increasingly popular. After chemical modification, starch paste viscosity and transparency are improved, along with its freeze–thaw and refrigeration stability, making it suitable as a food ingredient to improve noodle quality. Cooking time and cooking loss are key indicators used to evaluate noodle cooking quality [40]. The effects of OSA-esterified starch on noodle quality are summarized in Table 3. These results indicate that the addition of OSA-modified starch can significantly reduce noodle cooking time, demonstrating that noodles containing OSA-esterified starch require less energy for maturation than conventional noodles. This result is consistent with the lower gelatinization temperature and higher temperature sensitivity of OSA starch, as determined in the RVA analysis. This may be attributed to the large number of hydrophilic carboxyl groups introduced by OSA modification and the destruction of the starch surface morphology, which facilitates rapid water absorption into the noodles, thereby shortening their cooking time. Song et al. [41] investigated the effects of different starches on noodle quality and found that noodles cooked with corn starch containing the lowest amount of amylose required longer to cook than noodles prepared with acetylated corn starch. Shorter cooking times led to reduced exudate content during cooking, which also decreased the noodle loss rate. Additionally, owing to the enhanced hydrophilicity of OSA-modified starch, incorporating it into noodles increases their water absorption rate. Conclusively, these findings demonstrate that OSA-modified starch can serve as a promising food ingredient to enhance noodle quality, offering benefits such as reduced cooking times and improved texture.

## 4. Conclusions

OSA-modified starch is widely used as a thickener and emulsifier in food industries to regulate the quality of final products. In this study, we prepared two OSA-modified starches with varying degrees of substitution. The effects of OSA modification on the multiscale structure of starch were characterized, and the characterization results revealed that new functional groups were introduced into the internal structure of starch. Compared with native starch, esterified starch exhibited lower relative crystallinity, indicating that OSA esterification disrupted the ordered structure of the starch granules, damaged their integrity, and increased their surface roughness. Moreover, the incorporation of OSA-modified starch can regulate the retrogradation behavior of dough during heat treatment and reduce the final product’s hardness. Furthermore, the addition of OSA-modified starch shortened the cooking time of noodles and improved their water absorption rate. Therefore, the findings of this study highlight that OSA-modified starch has the potential to be used in fast edible starchy foods. Because OSA-modified starch has high apparent viscosity, future research should focus on modulating the fluidic food properties of OSA-modified starch by rationally designing its network and texture and validating its efficacy in dysphagia-friendly diets.

## Figures and Tables

**Table 1:** Pasting properties of wheat starch samples. Different lowercase letters indicate statistical significance at *p* < 0.05.

Samples	Peak Viscosity (cP)	Breakdown Viscosity (cP)	Final Viscosity (cP)	Setback Viscosity (cP)	Pasting Temperature (°C)
CWS	653 ± 14 ^c^	112 ± 13 ^c^	1044 ± 10 ^c^	503 ± 13 ^c^	93.90 ± 0.23 ^a^
EWS1	2239 ± 32 ^b^	275 ± 19 ^b^	3259 ± 23 ^a^	1295 ± 20 ^a^	62.60 ± 0.53 ^b^
EWS2	2511 ± 8 ^a^	627 ± 24 ^a^	2960 ± 18 ^b^	1076 ± 9 ^b^	60.00 ± 0.12 ^c^

**Table 2:** Mixolab parameters of mixed model flour. Different lowercase letters indicate statistical significance at *p* < 0.05.

Samples	C1/ (N·m)	C2/ (N·m)	C3/ (N·m)	C4/ (N·m)	C5/ (N·m)	C5 − C4/ (N·m)
CWSD	1.087 ± 0.010 ^b^	0.644 ± 0.003 ^c^	3.286 ± 0.014 ^a^	2.061 ± 0.024 ^b^	2.999 ± 0.015 ^a^	0.938 ± 0.387 ^a^
EWS1D	1.082 ± 0.021 ^b^	0.687 ± 0.024 ^b^	3.309 ± 0.012 ^a^	2.181 ± 0.009 ^a^	2.994 ± 0.009 ^a^	0.813 ± 0.004 ^b^
EWS2D	1.136 ± 0.013 ^a^	0.746 ± 0.019 ^a^	2.222 ± 0.009 ^b^	2.152 ± 0.010 ^a^	2.942 ± 0.005 ^b^	0.790 ± 0.008 ^b^

**Table 3:** Cooking characteristics of noodles. Different lowercase letters indicate statistical significance at *p* < 0.05.

Samples	Cooking Time (min)	Water Absorption Rate (%)	Cooking Loss Rate (%)
CWSD	8.6 ± 0.31 ^a^	220.3 ± 5.7 ^c^	11.9 ± 1.1 ^a^
EWS1D	7.2 ± 0.12 ^b^	245.7 ± 4.2 ^b^	9.1 ± 0.8 ^b^
EWS2D	6.1 ± 0.24 ^c^	257.8 ± 6.1 ^a^	8.9 ± 0.6 ^b^

## Data Availability

The original contributions presented in this study are included in the article. Further inquiries can be directed to the corresponding authors.
